# Experimental and Numerical Assessment of Fiber Orientation Effects in Biaxial Glass/Vinyl Ester Laminates

**DOI:** 10.3390/polym18020265

**Published:** 2026-01-19

**Authors:** Sultan Ullah, Arvydas Palevicius, Almontas Vilutis, Raul Fangueiro, Giedrius Janusas

**Affiliations:** 1 Department of Mechanical Engineering, Faculty of Mechanical Engineering and Design, Kaunas University of Technology, Studentų 56, LT-51424 Kaunas, Lithuania; arvydas.palevicius@ktu.lt (A.P.); giedrius.janusas@ktu.lt (G.J.); 2 Department of Engineering, UAB Poliplastas, Geliu St. 97, LT-53330 Kaunas, Lithuania; almontas@poliplastas.lt; 3 Fibrenamics, Institute of Innovation on Fibre-Based Materials and Composites, University of Minho, 4800-058 Guimarães, Portugal; rfangueiro@fibrenamics.com

**Keywords:** biaxial fabric composite, VARTM, thermoset, stacking sequence, mechanical properties, FEA

## Abstract

This study analyzes the mechanical behavior of a quasi-isotropic biaxial glass fiber–vinyl ester composite in a multiaxial stress condition and the effect of the orientation of the fibers. A ply structure was created through the process of vacuum infusion using six layers of biaxial fabric that were oriented to 15°. Tensile samples were isolated at 0, 15, 30, 45 and 90 degrees relative to the warp direction. It was found that strength and stiffness strongly depend on orientation, with maximum tensile strengths of 157.2 MPa at 90° and 125 MPa at 0°, and minimum tensile strengths 59.6 MPa at 15°, showing fiber and shear failures, respectively. MAT_124 underwent finite element analysis in LS-DYNA, and the results were excellent, with a difference of less than 1.5%. Three-point bending and Charpy impact tests indicated that flexural properties were lower at 15° and 90°, whereas off-axis orientations were generally better at impact energy absorption, although at 45°, binding sites were few and far between. The results have important implications for the design of laminates subjected to complicated loads.

## 1. Introduction

A composite material is formed by combining two or more constituent materials, resulting in properties that exceed those of the individual components. Reinforcement provides the mechanical strength to composite materials while the matrix bonds the reinforcement. Reinforcements have different forms: they can be woven, knitted, or braided, or even not, based on the method of manufacturing as well as the form of the fabric. Woven materials are made by the inter-weaving or intertwining of the strands of warp and weft, and their combination is termed a weave structure. The choice of application of a certain architecture in woven fabrics will be made in regard to the project requirements [1,2,3]. The usage of 3D fabrics has increasingly gained significance within the past two years, considering 3D fabrics have been found to be more helpful than 2D fabrics in damage resistance and delamination strength. Three-dimensional textiles, which combine warp, weft, and through the yarn thickness long with the z-tow direction to create another support fiber, as in 3D fabric structures, are being preferred in challenging applications in the transport, aerospace and construction industries [4,5,6].

Glass fiber-reinforced plastics (GFRPs) are extensively used in high-technology structural applications, including the aircraft and aerospace industries, owing to their favorable combination of high strength, stiffness, and durability [7,8,9,10]. Mechanical properties, in-plane structural mechanics, more traceability, and stability after higher-dimensional damage are not the only ways the structural engineering properties excel over those of nonwoven composites. In addition to the variability in woven composites, their evolution as fabrics, and their composition properties, numerous interesting variations in material behaviors and damage performance make them challenging to investigate and use. These aspects of fabrics have been the subject of much research owing to the mechanical and thermal attributes of woven composites and the convenience that woven composites provide in the manufacturing process. The most significant parameters of the areal density of the woven material are the number of fibers in a bundle and the density of the weave [11]. In order to demonstrate the direction dependence of the bending of a biaxial carbon fabric and that it can be nonlinearly defined at various angles of deflection, F. Yu et al. researched the bending of a fabric using carbon fabrics [12]. J. V. Viisainen et al., in their review, found wrinkling to be one of the major defects damaging the mechanical performance of composite structures [13]. Boisse et al., stated that when textiles show low bending stiffness, it results in slipping of fibers, and this increases the likelihood of wrinkling when making composites. This type of wrinkling introduces flaws, which undermine the mechanical properties of the resulting composite. These defects may disrupt both the normal distribution and edentulous orientation of load in between fibers in composites of biaxial glass woven fabrics and degrade the balanced in-plane mechanical performance of fibers, as well as making the structural performance less dependable [14]. Biaxial fabric is made of stitch yarn to enhance its mechanical properties. Flexural and tensile properties are also dependent on the order of stacking [15,16]. One of the most innovative techniques applied in the creation of composites is vacuum infusion, which has drawn significant interest. A common technique of producing high-performance composites is vacuum-assisted resin transfer molding, or VARTM. Being characterized by such advantages as low cost and reduced emission of volatile organic compounds (VOCs), VARTM became the viable option for the treatment of such materials. In addition to preventing the resin from being unevenly spread within the matrix, the strategy can be used to improve the mechanical and physical characteristics of the composite [17,18,19]. Woven composites have witnessed colossal application in fabric architecture in terms of tensile strength. Crimp ratio also has an effect on the mechanical characteristics. Weave level, geometry of the fabric, volume fiber content, structure of laminate, material system, etc., contribute significantly in the production of woven fabric composites. The fill and warp in a section are crossed one over the other in a fixed pattern of biaxial plain weaving [20,21]. Fabric composites showed more ductile-like behavior in the initial stage of development when compared to composite laminates in a nonlinear in-plane shear test when performing various load-carrying actions at the post-preparation stage. Unidirectional E-glass reinforcement has addressed the nonlinear stress–strain response to off-axis impact stress, and the composite performance was influenced by plasticity and damage. The regression option is employed to establish the variation in the overall mechanical performance of the change in the work-inflicted strain rate. A high strain rate can accelerate damage and also create damage in polymers. At the macro level, the loading direction affected the fiber bundle orientation, which in turn had an effect on the engineering properties [22]. It is also known that the elasticity and tenacity of broken composite materials decrease with increasing off-axis orientation, leading to a nonlinear stress–strain response under off-axis loading. Due to the interaction between tensile and shear forces, on-axis failure was observed to be less pronounced. The failed glass fiber specimens exhibit debonding between fiber bundles in the transition region and delamination between the layers. The correlation between global and local deformation behavior as a function of fiber orientation is established based on local strain responses measured at low-strain regions, high-strain regions, and the continuum scale deformation [23,24,25,26,27]. Fiber-reinforced polymer composites are also known for their brittle behavior [28,29]. Designers, therefore, consider the mechanical properties of woven fabric composites with respect to loading conditions and initial geometry. According to numerical simulations, damage behavior (transverse, matrix, and delamination) is used for the assessment of composites [30,31].

The MAT_124 material model in LS-DYNA is specifically designed for modeling materials that exhibit different behaviors in tension and compression. The model is particularly useful for materials that exhibit asymmetric stress–strain responses under different loading conditions. To date, this model has been successfully applied to modeling polymer composites with synthetic fibers. Appelsved modeled the mechanical behavior of thermoplastic polyamide reinforced with short glass fiber using several models, including MAT_124 [32]. Gurumurthy also employed the MAT_124 model among many adapted numerical models for composites to model a thermoset polymer composite with chopped glass fiber filler [33]. Other researchers used the MAT_124 model for modeling the mechanical properties of polypropylene (PP) with 16% talc as a filler [34]. However, to date, there is no evidence that this model has been applied to modeling polymer composites reinforced with natural fibers. The model is particularly useful for materials that exhibit asymmetric stress–strain responses under different loading conditions [35].

The discussion is specifically focused on biaxial woven glass/vinyl ester laminates and the limitations of traditional off-axis testing methods that inspire the laminate architecture and specimen-based orientation strategy adopted in the present study. In this experimental investigation, the mechanical behavior of biaxial glass/vinyl ester woven fabric composites with different layer orientations was examined, and it was established that tensile strength, flexural strength, and impact energy absorption under multiaxial stress conditions are strongly influenced by layer orientation. This study stands out from existing studies, which mainly involve unidirectional or single-layer composites, in that tensile strength, Charpy impact resistance, and three-point bending performance of a given composite are investigated with respect to biaxial fiber orientation and stacking sequence. This study employs a systematic analysis of the influence of off-axis orientation, supported by computational modeling (ANSYS suite R16.1 student version) of the experimental data. Although the influence of fiber orientation on woven glass fiber composites has been well reported, the literature on this subject is generally grounded on idealized on-axis/off-axis laminates or symmetrical stacking sequences. The existing literature on the relevant composite systems establishes the contextual background for this investigation [36,37,38,39,40]. On the contrary, the present study examines a quasi-isotropic biaxial glass/vinyl ester laminate produced through progressive 15-degree rotation of the fabric plies and evaluates the mechanical behavior of the fabric through specimen cutting alignment rather than ply rotation. Under this approach, all specimens are excised from a single laminate with identical material composition and curing history, allowing for the systematic isolation of orientation-sensitive deformation and failure mechanisms. Furthermore, the hybrid model of experiment–numerical also provides a renewed interpretation of the experimental–numerical interaction of stacking sequence, off-axis loading, and mixed-mode failure of the biaxial woven composite under multiaxial stress conditions. It is possible to expand the results in the future to develop predictive models and to further enhance the design of woven composites for more demanding applications.

## 2. Materials and Methods

### 2.1. Materials

In the present work, the synthetic reinforcement is a biaxial E-glass fabric with an areal density of 450 g/m^2^, purchased from Chongqing Yangkai Trade Co., Ltd. (Chongqing, China). Table 1 presents the detailed characteristics of the biaxial glass fabric. A vinyl ester resin supplied by Shanghai New Tianhe Resin Co., Ltd. (Shanghai, China) was used as the matrix material, and its main properties are summarized in Table 2. Curing was conducted using a cobalt-based accelerator and methyl ethyl ketone peroxide (MEKP), supplied by Sigma-Aldrich (St. Louis, MO, USA), and added at concentrations of 0.1 wt.% and 1 wt.%, respectively, in accordance with the resin manufacturer’s instructions. 

The composite fabrication matrix was a thermosetting vinyl ester resin, and the catalyst that was used to cure the resin was a normal hardener. VARTM composites with biaxial fabric use the resin due to its low viscosity, high toughness that facilitates easy impregnation and high adhesion of fibers and the matrix, biaxial architecture that allows equal in-plane stiffness and strength with high drapability of the laminates to be structurally efficient.

### 2.2. Fabrication of Composites

**Figure 1:**
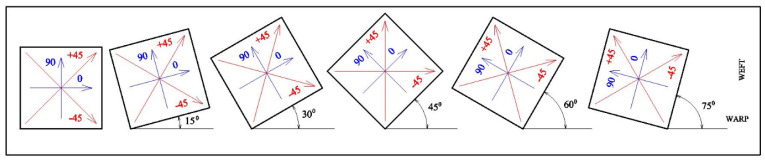
Scheme of cutting fabrics for lamination from a roll of biaxial E-glass. Blue color indicates stabilizing layers; red: main reinforcing layers.

**Figure 2:**
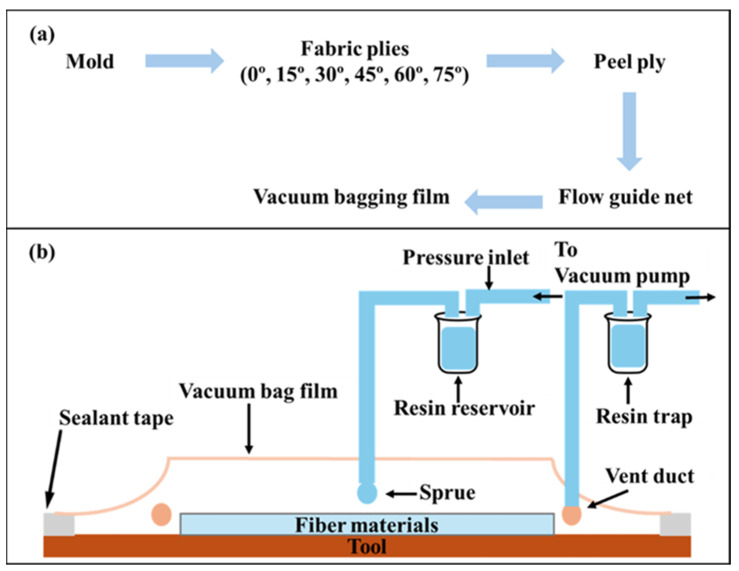
(**a**) Schematic showing use of vacuum bagging technique and (**b**) VARTM process.

**Figure 3:**
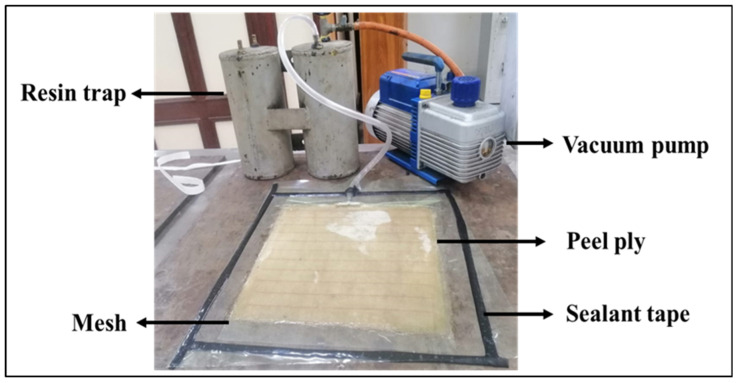
Biaxial composites fabrication process using VARTM.

The preparation of the tensile specimens involved a two-stage rotation process that first created a complex, quasi-isotropic laminate and then defined the testing orientation by cutting specimens at different angles from this single, uniform square plate, as shown in Table 3. The first stage involved the fabrication of the master laminate plate. This was achieved by stacking six individual pieces of the same 450 GSM biaxial glass fabric, each measuring 304 mm × 304 mm. The key step was the incremental 15° rotation of each fabric piece before it was added to the stack. The first piece of fabric was placed on the mold surface with its warp (0°) direction aligned as the reference (0° rotation). The second 304 mm × 304 mm piece was placed on top of the first, but it was rotated 15 degrees clockwise relative to the first piece. This process was repeated for all six pieces. The third piece was rotated 30 degrees clockwise, the fourth 45 degrees, the fifth 60 degrees, and the sixth and final piece was rotated 75 degrees clockwise. This 0–75-degree stacking sequence was selected to create a quasi-isotropically laminated polymer with a non-discrete distribution of fiber orientations. As a result, the effect of orientation can be systematically evaluated through specimen cutting direction without any influence from laminate composition or processing conditions. This stacking sequence resulted in a laminate in which the primary fiber orientations of the constituent fabrics were distributed through the thickness at 0°, 15°, 30°, 45°, 60°, and 75°, as shown in Figure 1. Figure 2 illustrates the schematic process of using vacuum-assisted resin transfer molding (VARTM), and Figure 3 shows the composite fabrication using the VARTM method.

This complex architecture was then consolidated via vacuum infusion at the Composite Center, Pakistan, using vinyl ester resin, and subsequently cured, resulting in a single, solid plate with a thickness of 3.3 mm and a highly quasi-isotropic internal structure. The laminate was allowed to cure for approximately 30 min at room temperature. Following the initial curing of the resin at room temperature, the prepared samples were transferred to an oven for post-curing of the vinyl ester resin at 100 °C for 1 h to achieve maximum cross-linking of the resin.

The second stage involved cutting the test specimens from this single, cured laminate plate. Instead of rotating the material during testing, the testing orientation was defined by the angle at which the specimen was cut from the plate.

The first specimen, coded S0, was cut from the plate with its longitudinal axis aligned perfectly with the original 0° warp direction of the first fabric piece. To obtain the other specimens, a cutting template for the 200 mm × 25 mm was rotated on the surface of the laminate plate. Sample S15 was cut by rotating the template 15 degrees clockwise from the S0 reference direction; sample S30, 30 degrees clockwise; S45, 45 degrees clockwise; S90, 90 degrees clockwise.

This specimen preparation plan is unlike the traditional off-axis testing schemes, where the laminate architecture is still maintained and the only effects of orientation are provided by the direction of cutting. Consequently, the difference in mechanical response can be fixed to the orientation of the fiber as opposed to loading, and, therefore, confounding factors that relate to laminate-to-laminate variations are avoided. In this way, tensile specimens were prepared, with the orientation of each layer of glass fabric relative to the direction of tension shown in Table 4.

### 2.3. Evaluation and Testing of Composites

#### 2.3.1. Mechanical Characterizations

To evaluate the performance of the fabricated composites, three mechanical characterization tests were conducted: tensile, three-point bending, and Charpy impact tests. A Universal Testing Machine (UTM) was used to perform the tensile and flexural tests, and a HIT50P pendulum impact tester was used to measure impact resistance at the Composite Center, Pakistan. The specimens had dimensions of 200 × 25 mm for tensile testing, 120 × 13 mm for three-point bending, and 100 × 10 mm for impact testing. The tests were performed according to ASTM D3039 [41], ASTM D7264 [42] and ISO 179-1 [43] standards. Analysis of the samples was carried out in Fibrenamics, Portugal.

Tensile and flexural tests were performed at room temperature, and the crosshead speed was maintained constantly at 2 mm/min. Tensile tests were conducted with various loading angles, and flexural tests were performed to determine the bending behavior of the composites. Impact strength and damage tolerance were measured in Charpy impact tests based on ISO 179-1 using a pendulum hammer with an impact energy of 5 J. Mechanical results are reported as mean value of all mechanical tests, and the error bars in the relevant figures represent the standard deviation calculated from a minimum of five specimens per configuration. Based on Classical Laminate Theory (CLT), mathematical dependencies of the strength and stiffness values obtained during experimental tests on the test orientation angle were established. A correlation analysis of experimental and modeled results was performed, and the coefficient of determination R^2^ and absolute percentage error (APE) were evaluated. To enable direct comparison of orientation dependence across different loading modes, the normalized anisotropy index (NAI = (max − min)/max) was calculated for all mechanical properties.

#### 2.3.2. Modeling of Composites

Numerical modeling was conducted to simulate the tensile tests and validate the model using the Ansys LS-DYNA Suite R16.1 student version against the experimental results, which is limited to a maximum of 128 × 10^3^ nodes/elements. The specimens were modeled using thick shell (TSHELL) elements (ELFORM = 6) within the finite element method. A coarser mesh of 2.5 mm × 1.25 mm was applied in the grip regions, while a uniform mesh of 1.25 mm × 1.25 mm was used elsewhere. The tensile specimen consisted of a total of 2800 elements (5922 nodes). Seven integration points through the specimen thickness were selected (NIP = 7). The elasto-viscoplastic TENSION_COMPRESSION_PLASTICITY (MAT_124) model was selected for modeling the composite specimens. During modeling, it was observed that the deformation curves quite accurately correspond to the deformational behavior of the materials and are consistent with the minimal number of experiments required for calibration of the MAT_124 model. In contrast, modeling with separate layers would require a larger number of tests to refine a greater number of parameters, along with more complex models whose calibration would demand significantly greater resources. Therefore, this modeling approach is acceptable, sufficiently accurate, and provides a foundation for more complex models.

The model parameters were determined from experimental curves and by matching numerical results with experimental data, and they are presented in Table 5.

Several parameters were applied to model material failure. First, the time-based delay parameter TDEL was specified in the MAT_124 model itself, which, in this case, is primarily intended to ensure numerical stability. To model plastic failure at the required time, an additional MAT_ADD_EROSION model was selected, with the corresponding parameters presented in Table 6. Since tensile specimens exhibit different failures, a two-failure-criterion condition was selected (NCS = 2). In addition to the maximum effective strain at failure (EFFEPS), the maximum principal strain at failure (MXEPS) criterion was also applied. In this case, failure occurs only when both criteria are satisfied, which better corresponds to actual composite failure. To achieve more accurate matching of the dynamic model results with the static test results, the failure parameter values in the MAT_ADD_EROSION model were slightly increased. Setting MXEPS > EFFEPS indicates that the material can withstand higher strains in the principal direction before failure compared to the multiaxial strain state. This reflects anisotropic material response and progressive damage sequence (loading → matrix damage (EFFEPS) → fiber damage (MXEPS) → complete failure). It is particularly appropriate for our natural fiber composites, where the matrix and interface are relatively weak compared to the fibers, leading to early matrix damage under multiaxial loading while the fibers can withstand higher strains in their principal direction.

##### Determination of the Main Composite Properties

Before performing numerical modeling, the material properties of the numerical simulations were calculated analytically and obtained experimentally. Laminate geometry, the properties of the constituent materials and the rule of mixtures (ROM) were used to compute the fiber volume fraction, the density of the composite, the Poisson ratio and the bulk modulus. The Young’s modulus used in the numerical simulations was the value calculated directly based on the original linear part of the experimental tensile stress–strain curves and was an input parameter.

According to the laminate thickness (3.3 mm), as measured, and the total fiber areal mass of six plies of 450 g·m^−2^ biaxial glass fabric, the fiber volume fraction was determined to be 31.7%. This value is similar to those of vacuum-assisted resin transfer molding (VARTM) composites. The rule of mixtures was used to determine the composite density, adjusted to reflect an average porosity level of 1.5%. The rule of mixtures was also used to calculate the ratio of Poisson that was assumed constant throughout all orientations since it was not directly measured.

Table 7 summarizes all the analytically determined homogenized composite properties that are utilized as numerical inputs.

## 3. Results and Discussion

### 3.1. Characterization of Composites

#### 3.1.1. Tensile Properties

**Figure 4:**
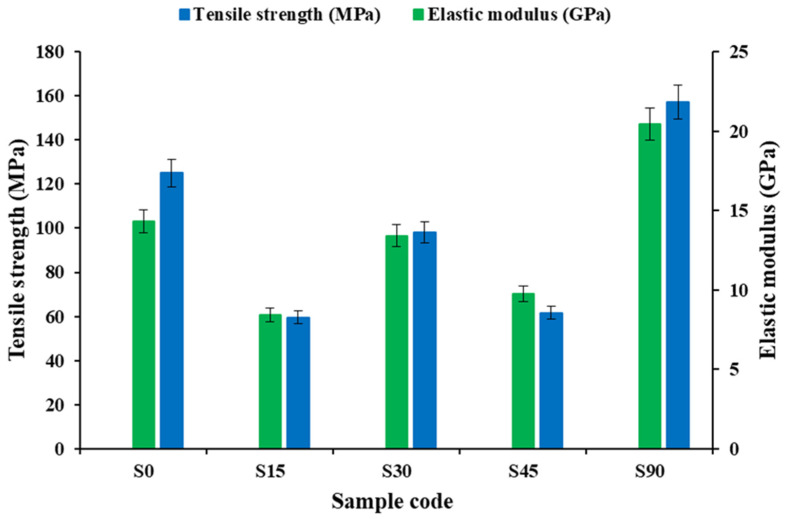
Experimental tensile strength and modulus of biaxial reinforced composites at various stacking sequences.

As shown in Figure 4, the experimental tensile strength and elastic modulus have a very strong correlation with the loading orientation, and the tensile behavior of the biaxial woven composites is largely governed by fiber alignment relative to the applied load. The obtained tensile strength and elastic modulus values are within the literature range of vacuum-infused biaxial and woven glass/vinyl ester composites, confirming that the laminate quality and mechanical response are representative and can be used in comparative analysis based on orientation [8,9]. The highest-performing specimens are S90 (UTS = 157.22 MPa; E = 20.45 GPa), indicating that the load process in them is effective when alignment is made between the most predominant direction of the yarns and the tensile axis. The S0 specimens (UTS = 125.07 MPa; E = 14.30 GPa) are also quite good, although clearly lower than those at 90°, which represents an asymmetry of the mechanical properties in the two major directions of the yarns. Anisotropy is typical of woven composites, which is more or less due to differences in yarn structure, crimp, nesting and local fiber volume distribution. The mechanical properties in the off-axis orientations are greatly degraded, particularly the 15- and 45-angle ones. The tensile strength and tensile modulus drop sharply to 15° (S15) to 59.63 MPa and 8.44 GPa, respectively, and the tensile strength is low (61.70 MPa), with a slight increase in the tensile modulus (9.77 GPa) at 45° (S45). This is pointed out by the efforts of these reductions in favor of a transference between fiber-controlled load bearing to matrix- or interface-controlled deformation. The large shear stresses at the crossover points with yarns and in the matrix created by off-axis loading promote the onset of damage in the matrix and inter-yarn and interfacial debonding, which accelerates nonlinear deformation and premature failure. The response at 30° (S30) is non-monotonic, in which a partial recovery of both tensile strength (98.15 MPa) and modulus (13.42 GPa) of the responses at 15° and 45° is observed. This behavior suggests better load sharing between the axial and shear transfer behaviors, with the two yarn systems of fabric contributing more to the load resistance, as compared to shearing-dominated behavior, which is highly deforming at high off-axis angles. This highlights the influence of the peculiarities of the fabric, i.e., yarn waviness and interlacing constraints, to establish the off-axis tensile behavior.

Overall, the test results indicate that tensile strength and stiffness reach their highest levels when the yarns lie along a major axis and particularly at 90° (S90), and the off-axis direction indicates the failure of fiber efficiency due to shear-related damage mechanisms. The standard deviation of the data is relatively small, which implies that the experiments can be repeated with high repeatability; however, the large orientation sensitivity would imply that any slight variation in the cut angle, heterogeneity of the weave, etc., can cause some significant off-axis performance change. These findings confirm the cut orientation as one of the most critical design factors in biaxial woven composites, and middle ranges have lower performance recovery because they have better multi-yarn load sharing mechanisms.

All the specimens were extracted from the same laminate with identical fiber volume fraction, density, and processing conditions; therefore, normalization of the mechanical properties in terms of the density or fiber content would not have any effect on the comparative trends and, as such, is not necessary for the objectives of this study.

The proposed failure mechanisms are determined based on the nature of the mechanical response and the existing literature, rather than on direct microscopic observation.

#### 3.1.2. Impact Properties

**Figure 5:**
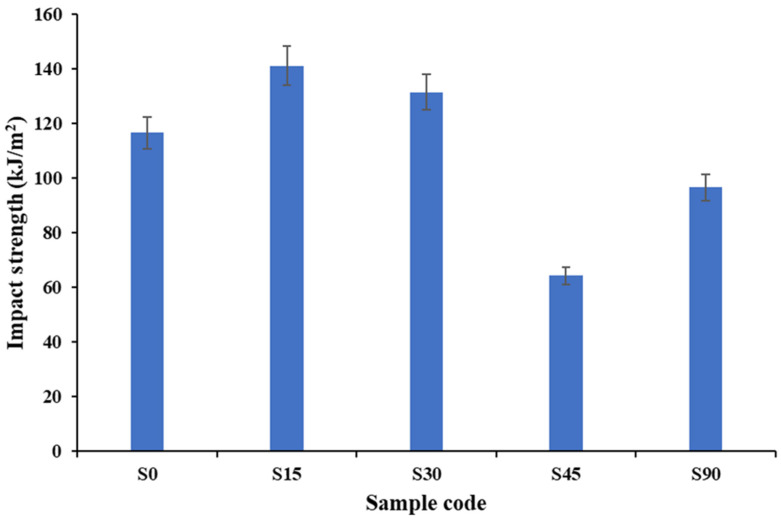
Experimental impact strength and modulus of biaxial reinforced composites at various stacking sequences.

As Figure 5 (experimental data) demonstrates, the effect of fiber orientation on the impact strength of the biaxial woven composites is strong. The 15° and 30° orientations (S15 and S30) have the highest impact energies (141.10 and 131.47 kJ/m^2^, respectively), followed by the 0° orientation (116.54 kJ/m^2^), indicating that more energy is dissipated when both fiber systems are functionally engaged during impact. Near-axis orientations promote fiber expansion, matrix deformation, and inter-yarn friction. These mechanisms slow crack initiation and encourage partial rather than catastrophic failure. Conversely, the 45-degree specimen (S45) has the lowest impact strength (64.24 kJ/m^2^), which is characteristic of a shear-dominated failure mode; inter-yarn sliding and matrix shear cracking are easy to generate, and this puts a constraint on energy uptake. It exhibits intermediate impact resistance (96.58 kJ/m^2^), indicating lower fiber involvement and more dependence on processes of damage, over which the matrix has control. Impact performance is maximized at near-axis configurations because of enhanced load redistribution and crack-bridging effects, whereas off-axis combinations, especially at S45, show reduced impact tolerance because of shear-induced damage.

The fact that all the specimens were extracted out of the same laminate, and, hence, they share the same thickness and volume, makes the discussion of the absorbed impact energy a relative one to highlight the behavior, which is dependent on orientation, rather than normalized values.

#### 3.1.3. Flexural Properties

**Figure 6:**
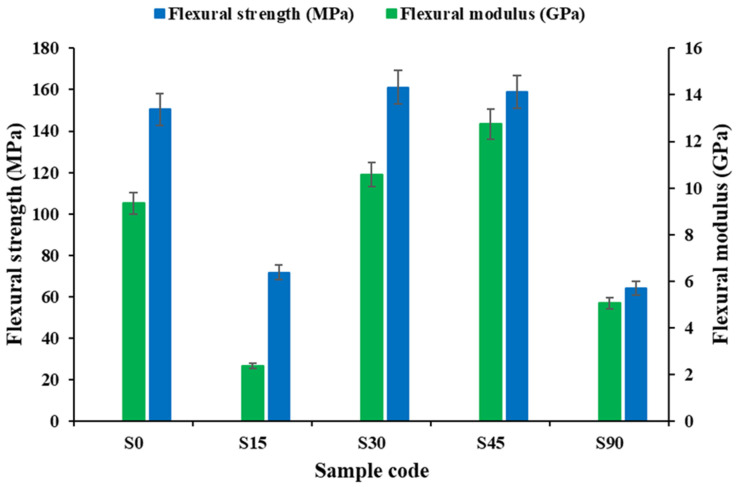
Experimental flexural strength and modulus of biaxial reinforced composites at various stacking sequences.

Figure 6 (experimental data) demonstrates that the flexural response of the biaxial glass-woven composites is strongly governed by fiber orientation, consistent with the orientation sensitivity observed under tensile and impact loading. Specimens oriented at 0° (S0), 30° (S30), and 45° (S45) exhibit the highest flexural strength and modulus (≈140–160 MPa), indicating efficient load sharing between fibers on the tensile and compressive sides during bending, which results in stiff behavior and abrupt, fiber-dominated failure. In contrast, the 15° (S15) and 90° (S90) orientations show significantly reduced flexural strength (≈60–70 MPa) and stiffness, reflecting inefficient fiber alignment with the principal bending stresses and a greater contribution from matrix deformation, interfacial debonding, and delamination. These off-axis configurations exhibit more progressive deformation due to increased matrix participation and interfacial damage but with reduced bending load-carrying capacity. Overall, the results suggest that flexural performance depends on the orientation of the fibers relative to the direction of the applied bending stress, with alignments that are more consistent with the major axes of the fibers giving higher flexural strength and stiffness, and misalignment orientations are linked to lower flexural efficiency.

#### 3.1.4. Experimental Tensile Data Validation by Numerical Simulation and Models

**Figure 7:**
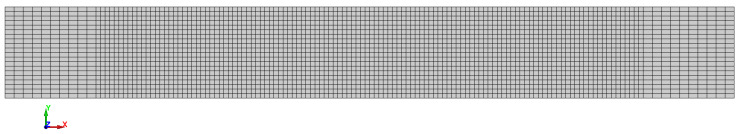
Finite element mesh of the tensile specimen 200 mm × 25 mm × 3.3 mm (gauge length L_g_ = 150 mm).

**Figure 8:**
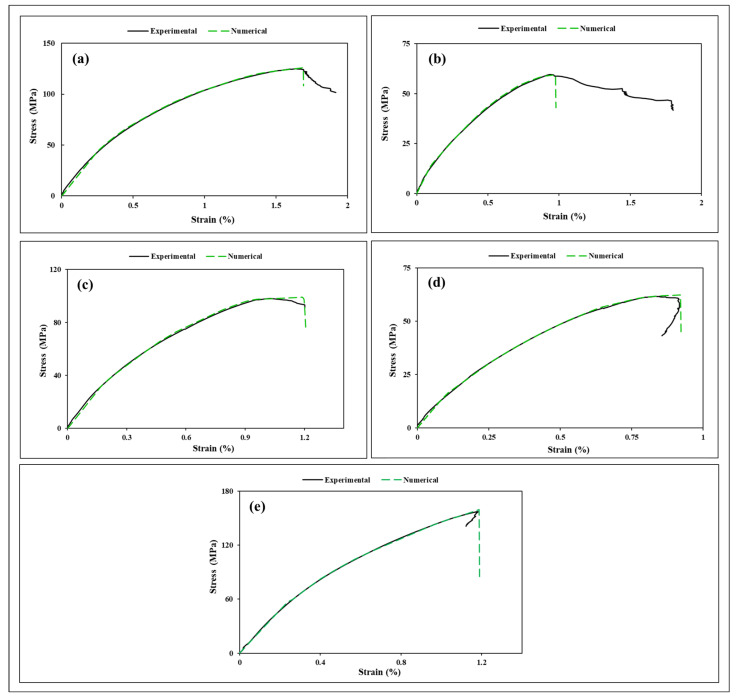
The experimental and numerical tensile test curves: (**a**) S0; (**b**) S15; (**c**) S30; (**d**) S45; and (**e**) S90.

A tensile simulation was performed, and its schematic representation is illustrated in Figure 7. The experimental and numerical tensile test curves are presented in Figure 8. As can be seen, all composite experimental tensile stress–strain curves (black solid lines) coincide with numerical curves (green dashed lines) with sufficient accuracy, and the shape of the deformation curves is similar until the peak force. Following a very short linear region, the curves bend and increase steadily until maximum load is reached.

From the tensile curves (Figure 8), it can be observed that while all specimens exhibit a degree of brittle behavior, their post-peak response varies significantly. Samples S30, S45, and S90 display largely brittle behavior, characterized by a sharp, distinct peak in stress followed by an abrupt drop at the point of fracture. In contrast, S15 exhibits a more ductile response with a long tail, arising from distributed shear-dominated damage and progressive fiber rotation, which enable stable load redistribution and prevent abrupt failure despite low peak strength. Meanwhile, S0 displays semi-brittle behavior with a shorter, more pronounced tail. Regarding the strain to failure, the experimental curves reveal a narrow range of values, approximately between 1.0% and 2.0%. Interestingly, the trend in ductility does not mirror the trend in tensile strength. For instance, sample S15 (Figure 8b), while exhibiting the lowest peak strength (~60 MPa), demonstrates one of the highest strain-to-failure effects (composite S15), exceeding 1.5%, due to its progressive, mixed-mode failure. Conversely, sample S45, also with low strength, fails at the lowest strain (composite S45), around 0.9%, indicating a more catastrophic, shear-dominated fracture. The high-strength samples, S0 and S90, show moderate and similar strains to failure (composite S0, S90), consistent with their brittle, fiber-dominated failure modes. This decoupling of strength and ductility highlights the complex interplay between fiber orientation and the resulting failure mechanisms in this quasi-isotropic laminate. The negative tail observed in the tensile curves of S45 and S90 indicates a loss of load-carrying capacity combined with residual deformation under displacement control, rather than true compressive material behavior. After peak load, extensive matrix cracking, fiber–matrix debonding, and ply sliding occur, causing the specimen to continue deforming while the measured force drops and briefly reverses sign due to grip compliance, specimen recoil, and shear-dominated unloading. In S45, this effect reflects abrupt shear band formation and unstable failure, whereas in S90, it is associated with progressive fiber bridging and delayed load transfer collapse, producing a smoother post-peak response. The negative tail, therefore, signifies structural softening and damage-controlled unloading, not the compressive strength of the laminate.

It is important to note that the MAT_124 model is well suited for capturing this overall response. This model is designed to simulate elastic–plastic behavior up to a failure criterion, after which elements can be eroded. It accurately reflects the material’s behavior up to the maximum force, particularly for the brittle or semi-brittle failures seen in S0, S30, S45, and S90. The limitations of the model, such as its inability to predict long post-peak ductile tails, become most apparent when comparing with the S15 curve. However, even for S15, the model successfully predicts the peak stress and initial stiffness with high accuracy. The excellent agreement between the experimental curves and the numerical simulations across all sampling orientations validates the use of the homogenized material properties within the model for predicting the key mechanical performance metrics. The curves in Figure 8 differ significantly in their peak stress and initial slope (modulus), revealing a strong dependence on the sampling orientation relative to the internal structure of the laminate. A clear pattern emerges when grouping the samples by their mechanical response. Samples S0 and S90 form a distinct group with the highest peak tensile strength (125.1 MPa for composite S0 and 157.2 MPa for composite S90) and the steepest initial modulus. In contrast, samples S15 and S45 form a second group with the lowest peak strength (59.6 MPa for composite S15 and 61.7 MPa for composite S45). Sample S30 stands as an intermediate case, showing a significant recovery in strength (98.2 MPa for composite S30) compared to the low-strength group. This nonlinear trend indicates that the mechanical response is not a simple function of the cutting angle but is governed by the complex interaction of the multiple fiber orientations within the laminate.

A mechanistic explanation reveals that the loading direction dictates whether the failure is fiber-dominated or shear-dominated. For samples S0 and S90, the load is carried directly by fibers aligned with the loading direction. This results in a high-stiffness, high-strength response where failure is abrupt and governed by the fracture of the glass fibers themselves. The similarity between S0 and S90, despite their different cutting angles, is explained by the quasi-isotropic nature of the laminate. The stack sequence ensures that loading in the 0° or 90° direction engages a similar arrangement and density of load-bearing fibers.

In contrast, the failure of sample S45 is shear-dominated. At a 45° cutting angle, no fibers are effectively aligned with the tensile load. The applied stress is resolved into shear stresses within the matrix and at the fiber–matrix interfaces. Because the vinyl ester matrix is significantly weaker and less stiff than the glass fibers, the overall composite exhibits a much lower strength and fails via matrix cracking and fiber–matrix debonding. The surprisingly low strength of sample S15, even lower than the shear-dominated S45 case, can be attributed to its specific ply configuration ([15/60/75/−60]), which may create a particularly unfavorable and inefficient load path, leading to premature failure under a mixed-mode of tension and shear. The more ductile post-peak behavior of S15 suggests a more progressive failure process, likely involving sequential fiber bundle pull-out and extensive matrix cracking, which the numerical model simplifies into a single failure event. Although the total volume of fibers is equal, the synergistic interaction and stacking sequence of the various ply orientations within each sample dictate the overall strength. The performance is not determined by the mere presence of certain ply angles but by how the entire stack of plies works together to resist the applied load, starting from the outermost surfaces.

**Figure 9:**
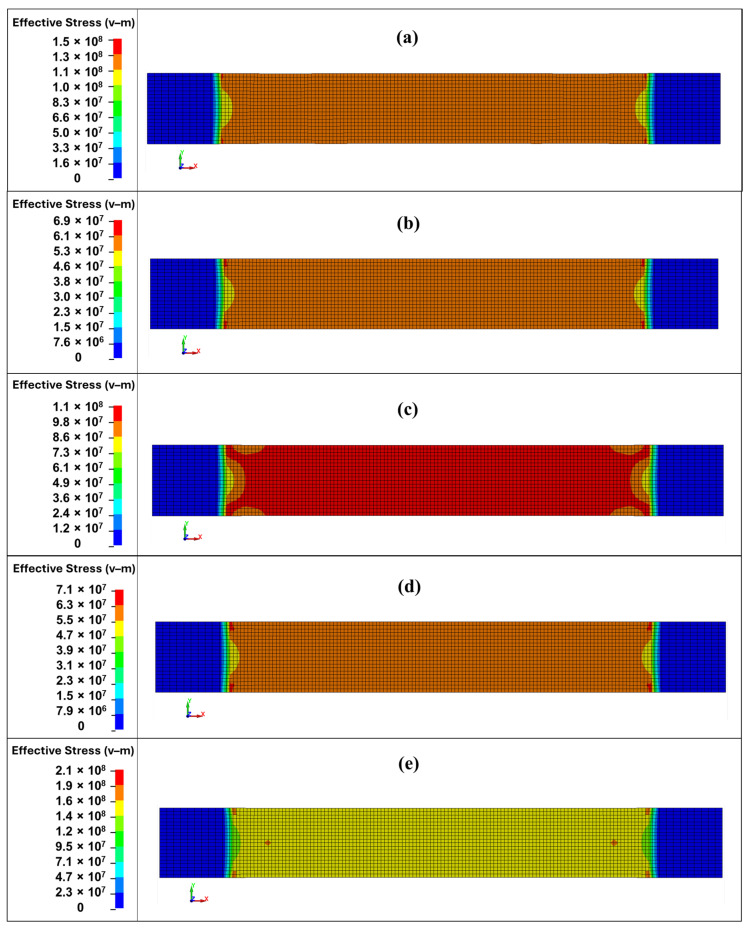
Equivalent von Mises σ^VM^ stresses in SI units Pa at 0.01 ms before the start of failure (start of element deletion) after dynamic tensile test simulation: (**a**) S0 (16.88 ms); (**b**) S15 (9.73 ms); (**c**) S30 (11.96 ms); (**d**) S45 (9.18 ms); and (**e**) S90 (11.86 ms).

**Figure 10:**
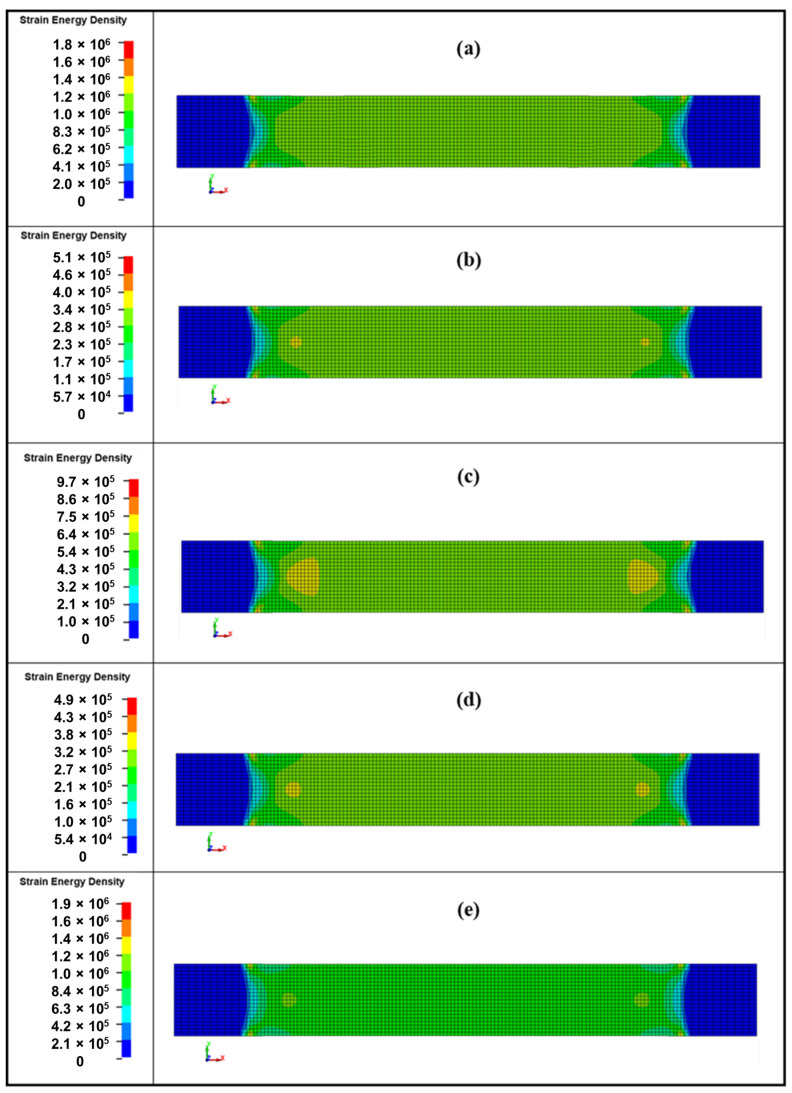
Strain energy density in SI units J·m^−3^ at 0.01 ms before the start of failure (start of element deletion) after dynamic tensile test simulation: (**a**) S0 (16.88 ms); (**b**) S15 (9.73 ms); (**c**) S30 (11.96 ms); (**d**) S45 (9.18 ms); and (**e**) S90 (11.86 ms).

The equivalent von Mises stresses for tensile specimens are illustrated in Figure 9, while Figure 10 presents the strain energy density (SED) for the same tensile specimens. The contour plots are supposed to give qualitative information of the redistribution and orientation-dependent deformation of stress but not the quantitative damage information.

From the numerical tensile results [44,45], it can be observed that localized stress concentration occurs at the specimen grip ends, with stresses distributed symmetrically at both ends. In the numerical model, the ends are ideally constrained, whereas the actual constraint differs to some extent. Moreover, the actual specimen exhibits fiber waviness, resin inhomogeneities, surface defects, microcracks, non-uniform matrix–fiber adhesion, and regions of varying specimen thickness. In the numerical model, the specimen fails at either the fixed or moving end, whereas the actual specimen may fail at the center if a weaker region develops there, despite the fact that stresses at the ends may be somewhat higher. Based on the deformation–strain tensile curves and their agreement with the numerical results, when appropriate composite properties are assigned, the mechanical behavior of such composites can be estimated with sufficient accuracy.

From the von Mises stress and strain energy density (SED) distributions, a clear transition in failure mechanisms based on sampling orientation is evident. For samples S90 and S0, which exhibit the highest tensile strength, the von Mises stresses are the highest (peaks of ~214 MPa and ~150 MPa, respectively) and concentrated in the central gauge section. Correspondingly, the SED is also high and localized, with values approaching 1.0 MJ·m^−3^. This indicates that the load is carried efficiently by fibers aligned with the loading direction, storing significant energy before a sudden, brittle, fiber-dominated tensile failure.

In contrast, when analyzing sample S45, which exhibits the lowest strength, the peak von Mises stress is significantly lower (~72 MPa). The stress distribution is more uniform across the gauge width, and the SED is also low and widely distributed. This indicates that no fibers are effectively loaded in tension, and the material fails prematurely. The low SED value (0.49 MJ·m^−3^) confirms that little energy is absorbed before failure. This behavior is characteristic of a shear-dominated failure, where the ability of the matrix to transfer load between fibers is the limiting factor, leading to matrix cracking and fiber–matrix debonding at 45° to the load path.

The intermediate samples, S15 and S30, exhibit a mixed-mode response. Interestingly, S15 shows the lowest tensile strength (60 MPa), even lower than the shear-dominated S45 case (62 MPa). Its von Mises stress and SED are low and indicate an inefficient load transfer, likely due to its specific ply orientation ([15/60/75/−60]) being particularly unfavorable. Sample S30, however, shows a significant recovery in tensile strength (~100 MPa), with higher peak von Mises stress and SED than S15. This suggests that its ply configuration ([30/75/60/−75]) allows for a more effective combination of fiber tension and matrix shear, resulting in a stronger mixed-mode failure. The combination of von Mises stress and SED maps reveals how the sampling orientation fundamentally alters the failure mode of this quasi-isotropic laminate. The S0/S90 orientations exhibit high, localized stress and SED, indicating efficient load transfer to fibers and a brittle, fiber-tension failure. The S45 orientation shows low, distributed stress and SED, confirming a weak, shear-dominated failure governed by the matrix. The intermediate S15 and S30 samples demonstrate a complex mixed-mode failure, where the specific ply angles dictate the balance between fiber and matrix load bearing, resulting in a nonlinear strength trend. Mechanistically, this behavior is explained by resolving the tensile load into axial and shear components relative to the various fiber orientations within the laminate, which directly controls the stiffness, strength, and ultimate failure pathway.

When evaluating the maximum tensile stress (Table 8), the numerical results demonstrated an exceptionally high degree of accuracy, with differences from the experimental values confined to a minimal range of 0.3–1.4% across all composites. This remarkably close correlation serves as a powerful validation for the LS-DYNA model utilizing the MAT 124 material definition. It confirms that the calculated homogenized properties effectively capture the bulk mechanical response of the complex laminate and that the model is well suited to simulate its predominantly brittle failure mode. The minor discrepancies are well within the expected tolerance for such simulations and can be attributed to the idealizations inherent in the numerical model, such as perfectly homogeneous material and boundary conditions.

In addition to determining the behavior of anisotropy, the results reveal the non-monotonic behavior of strength development and the failure processes that are orientation-dependent, and one cannot be preferred using fiber alignment. In particular, the low strength with a high ductile nature of the S15 structure and regeneration of the partial strength of the S30 demonstrate the significance of the ply interaction and the stacking synergy in quasi-isotropic biaxial laminates. These findings show that the ply orientations are not important in determining the mechanical performance of the laminate, but the collective behavior of the laminate is.

#### 3.1.5. Quantitative Analysis of Orientation-Dependent Mechanical Properties

The mechanical performance of the laminate is profoundly governed by the loading mode, resulting in distinct and contrasting anisotropic behaviors. Table 9 presents these anisotropy metrics along with the corresponding maximum and minimum values and their orientations. Quantified by the normalized anisotropy index (NAI), tensile strength (62.1%) and flexural stiffness (81.4%) exhibit the highest sensitivity to orientation, while impact strength (54.5%) is the least anisotropic. This extreme sensitivity in flexural stiffness, the highest of all properties, is a direct consequence of bending mechanics, where performance is dominated by the material response at the outermost surfaces. The vast difference between the maximum modulus at 45° (12.74 GPa) and the minimum at 15° (2.37 GPa) demonstrates that the orientation of these surface plies is paramount; a 15° test orientation angle renders them exceptionally inefficient, causing a near-total collapse of the laminate’s rigidity. In stark contrast, impact strength peaks at 15° because this orientation promotes synergistic energy dissipation mechanisms like fiber pull-out, rather than efficient load transfer. This fundamental divergence reveals that the design of this laminate is a critical trade-off, requiring a 0°/90° alignment for maximum static strength and stiffness or an off-axis alignment for superior impact resistance.

**Figure 11:**
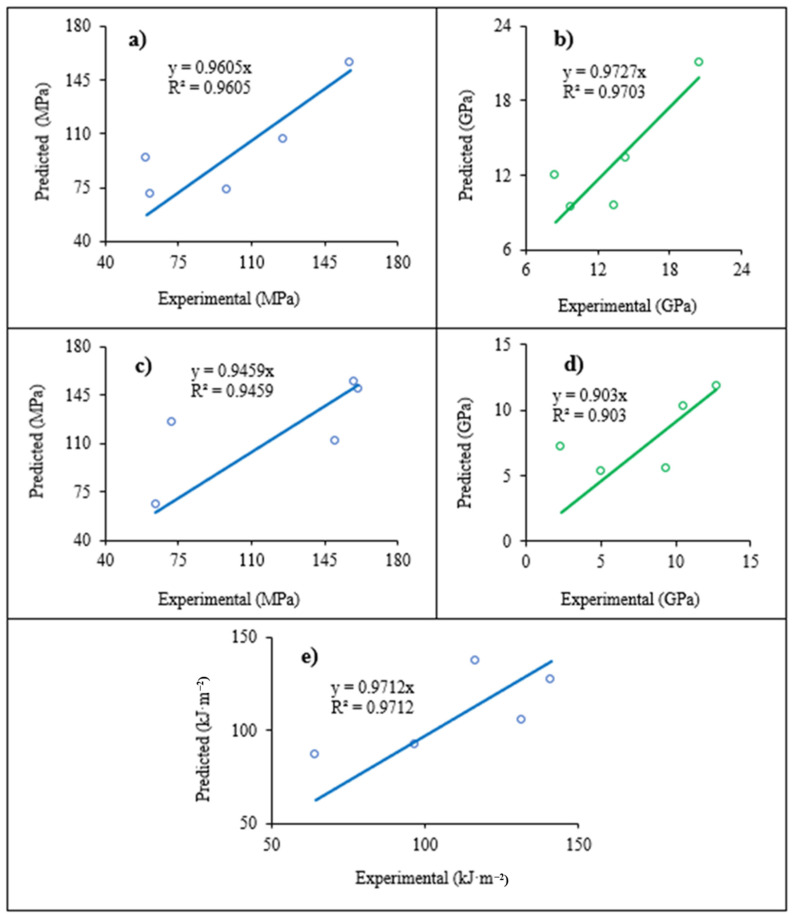
Comparison between the experimental data and the prediction obtained by the proposed model: (**a**) tensile strength; (**b**) tensile modulus; (**c**) flexural strength; (**d**) flexural modulus; and (**e**) Charpy impact strength.

To capture these complex relationships, predictive equations were developed and validated against experimental data. The specific equations for tensile strength, tensile modulus, flexural strength, flexural modulus, and impact strength, along with their statistical validation, are presented in Table 10. The corresponding comparative data are shown in Figure 11.

While the models demonstrate a strong overall correlation with experimental results (R^2^ > 0.9), their accuracy is highly dependent on the property and test orientation. The models struggle most at the 15° orientation for tensile and flexural properties, where absolute percentage errors (APEs) reach as high as 58.4% for strength and a remarkable 201.8% for flexural modulus. This highlights the challenge of modeling the complex shear-dominated failure and stiffness collapse that occurs at this critical angle. Conversely, the models perform exceptionally well at the 90° orientation, with APE values below 2.5% for tensile and flexural strength. The Charpy impact model showed the most consistent performance, with a relatively low average APE of 17.5%, while the flexural modulus model had the highest average APE (51.4%), reflecting the difficulty in predicting its extreme anisotropy.

## 4. Conclusions

This systematic study examining the mechanical behavior of biaxial glass/vinyl ester laminates under multiaxial loading conditions demonstrated that angle-pattern layup is an important factor in the tensile, flexural and impact behavior. The experimental–numerical approach offers some insight into how fiber orientation depends on stiffness, stress redistribution, damage formation, and failure modes.

On-axis laminates (S0 and S90) exhibited better tensile strength, elastic modulus and linear stress/strain behavior because they provide an effective transfer of the axial loads with the aid of outer plies, which are oriented in the direction of loading. These orientations failed in fiber in a brittle manner, having a high concentration of stress and strain energy. On the other hand, the S45 laminate was the weakest among the rest of the laminates, in which the outer plies of ±45° were biased towards shear-oriented matrix cracking and interfacial debonding, disallowing effective utilization of the inner plies. The intermediate orientations (S15 and S30) showed mixed-mode failure behavior, which is dictated by the ratio of the axial fiber loading to shear deformation regulated by the outer-layer orientation.

The impact testing revealed off-axis laminates were more efficient in energy absorption since the fiber network was more engaged because of crack deflection and enhanced fiber–matrix cross-linkage, resulting in progressive damage. Some experiments that used the LS-DYNA and mat material model MAT 124 showed that the experimental tensile result was close to the numerical simulations at the expense of 0.3–1.4%, which is an affirmation of the plausibility of the homogenized modelling procedure. From a design perspective, near-axis orientations are suited for stiffness- and load-bearing applications, whereas off-axis or intermediate orientations favor damage tolerance and energy absorption. These findings provide practical guidance for tailoring fiber orientation and stacking sequence in biaxial woven laminates to meet specific structural requirements.

Generally, the findings show that the significant design variables to tailor the composite performance are the fiber orientation and stacking sequence. It will be extended to fatigue loading, environmental aging and hybrid laminate architecture in the future.

## Figures and Tables

**Table 1:** Specifications of biaxial fabric.

Construction	Areal Density (oz/yd^2^)	Tolerance (%)	Material
0°	1.29	±4.95	E-glass 68 tex
+45°	2.24	±4.95	E-glass 300 tex
90°	1.27	±4.95	E-glass 68 tex
−45°	2.24	±4.95	E-glass 300 tex
Stitching	6	±4.95	Polyester 83 dtex

**Table 2:** Properties of vinyl ester resin.

Properties	Value
Density	1.1–1.2 g/cm^3^
Tensile strength	≈69 MPa
Tensile modulus	3.0–3.4 GPa
Tensile elongation at break	≈3.2%
Flexural strength	120–135 MPa
Flexural modulus	3.2–3.5 GPa
Glass transition temperature, T_g_	82–104 °C

**Table 3:** Details of fabricated composite stacking sequence for mechanical testing.

Sample Code	Reinforcement	Stacking Sequence	Test Orientation (°)
S0	6 layers of biaxial glass fabric	0°/15°/30°/45°/60°/75°	0°
S15	6 layers of biaxial glass fabric	0°/15°/30°/45°/60°/75°	15°
S30	6 layers of biaxial glass fabric	0°/15°/30°/45°/60°/75°	30°
S45	6 layers of biaxial glass fabric	0°/15°/30°/45°/60°/75°	45°
S90	6 layers of biaxial glass fabric	0°/15°/30°/45°/60°/75°	90°

**Table 4:** Absolute ply orientations in the tensile specimen coordinate system.

Piece (Net Rotation Angle)	Layer 1 (0°)	Layer 2 (+45°)	Layer 3 (90°)	Layer 4 (−45°)
S0 specimen:				
1 (0°)	0°	+45°	+90°	−45°
2 (15°)	+15°	+60°	+105°	−30°
3 (30°)	+30°	+75°	+120°	−15°
4 (45°)	+45°	+90°	+135°	0°
5 (60°)	+60°	+105°	+150°	+15°
6 (75°)	+75°	+120°	+165°	+30°
S15 specimen:				
1 (−15°)	−15°	+30°	+75°	−60°
2 (0°)	+0°	+45°	+90°	−45°
3 (+15°)	+15°	+60°	+105°	−30°
4 (+30°)	+30°	+75°	+120°	−15°
5 (+45°)	+45°	+90°	+135°	0°
6 (+60°)	+60°	+105°	+150°	+15°
S30 specimen:				
1 (−30°)	−30°	+15°	+60°	−75°
2 (−15°)	−15°	+30°	+75°	−60°
3 (0°)	+0°	+45°	+90°	−45°
4 (+15°)	+15°	+60°	+105°	−30°
5 (+30°)	+30°	+75°	+120°	−15°
6 (+45°)	+45°	+90°	+135°	0°
S45 specimen:				
1 (−45°)	−45°	+0°	+45°	−90°
2 (−30°)	−30°	+15°	+60°	−75°
3 (−15°)	−15°	+30°	+75°	−60°
4 (0°)	0°	+45°	+90°	−45°
5 (+15°)	+15°	+60°	+105°	−30°
6 (+30°)	+30°	+75°	+120°	−15°
S90 specimen:				
1 (−90°)	−90°	−45°	0°	−135°
2 (−75°)	−75°	−30°	+15°	−120°
3 (−60°)	−60°	−15°	+30°	−105°
4 (−45°)	−45°	0°	+45°	−90°
5 (−30°)	−30°	+15°	+60°	−75°
6 (−15°)	−15°	+30°	+75°	−60°

**Table 5:** MAT_124 model parameters for composite tensile tests.

Parameter	Name	Unit	S0	S15	S30	S45	S90
RO	Mass density	kg·m^−3^	1612.5	1612.5	1612.5	1612.5	1612.5
E	Young’s modulus	MPa	15,000	12,200	15,000	13,500	20,500
PR	Major Poisson’s ratio		0.31	0.31	0.31	0.31	0.31
TDEL	Min time step size for element deletion	s	1 × 10^−10^	1 × 10^−10^	1 × 10^−10^	1 × 10^−10^	1 × 10^−10^
EC	Young’s modulus for compression	MPa	15,000	12,200	15,000	13,500	20,500
RPCT	Scaling factor between E and EC		0.5	0.5	0.5	0.5	0.5
PC	Compressive mean stress (pressure)	MPa	6.00	4.00	6.0	4.0	7.80
PT	Tensile mean stress (pressure)	MPa	6.00	4.00	6.0	4.0	7.80
K	Bulk modulus	MPa	13,160	10,700	13,160	11,840	17,980

Source: author’s own elaboration.

**Table 6:** MAT_ADD_EROSION model parameters for composite tensile numerical tests.

Parameter	Name	S0	S15	S30	S45	S90
EFFEPS	Max effective plastic strain at failure	0.0210	0.0145	0.0170	0.0133	0.0168
MXEPS	Max principal strain at failure	0.0253	0.0150	0.0170	0.0133	0.0168

**Table 7:** Analytically determined homogenized composite properties used as input parameters for numerical simulations.

Property	Symbol	Value	Determination Method
Fiber density	ρf	2580 kg·m^−3^	Manufacturer data
Matrix density	ρm	1200 kg·m^−3^	Manufacturer data
Total fiber areal mass (6 plies)	—	2.700 kg·m^−2^	Fabric areal density
Laminate thickness	t	3.3 mm	Experimental measurement
Fiber volume fraction	V_f_	0.317 (31.7%)	Areal mass & thickness calculation
Matrix volume fraction	V_m_	0.683	1 − V_f_
Composite density (ROM)	ρ_lam_	1637.5 kg·m^−3^	Rule of mixtures
Porosity-adjusted density	RO	1612.5 kg·m^−3^	1.5% porosity correction
Poisson’s ratio (composite)	υ_c_	0.31	Rule of mixtures
Young’s modulus	E	Orientation-dependent	Experimental tensile curves
Bulk modulus	K	See Table 5	Derived from E and υ

**Table 8:** Comparison of maximum stress σ_max_ results (MPa) and elongation ε_max_ (%) obtained by experiment and numerical tensile modeling.

Sample	Maximum Stress σ_max_ (MPa)			Maximum Strain ε_max_ (%)		
	Experiment	Numerical	Diff. %	Experiment	Numerical	Diff. %
S0	125.0719	125.9509	0.70	1.63	1.68	2.97
S15	59.6379	59.4801	−0.26	0.94	0.97	3.09
S30	98.1581	99.0596	0.92	1.14	1.18	3.03
S45	61.7064	62.3332	1.02	0.85	0.90	5.55
S90	157.2297	159.3840	1.37	1.181	1.186	0.42

**Table 9:** Anisotropy metrics and directional dependence of mechanical properties.

Property	Max Value	Min Value	NAI	Interpretation
Tensile strength (MPa)	157.22 (S90)	59.63 (S15)	0.621	62.1% of the maximum tensile strength is lost at the worst-case test orientation (15°), indicating strong directional dependence where off-axis loading results in matrix-dominated failure rather than efficient fiber load transfer.
Tensile modulus (GPa)	20.45 (S90)	8.44 (S15)	0.587	58.7% of the maximum stiffness is lost at the worst-case test orientation (15°). This is nearly as high as strength, confirming that stiffness is also highly dependent on efficient fiber load transfer.
Flexural strength (MPa)	161.15 (S30)	64.10 (S90)	0.602	60.2% of the maximum flexural strength is lost at the worst-case test orientation (90°). This anisotropy level is similar to tensile strength and higher than impact energy, indicating that bending performance is highly sensitive to the orientation of both the top and bottom plies, which must work together to resist compression and tension respectively.
Flexural modulus (GPa)	12.74 (S45)	2.37 (S15)	0.814	81.4% of the maximum stiffness is lost at the worst-case test orientation (15°). This is the highest anisotropy among all properties measured, confirming that flexural stiffness is exceptionally sensitive to the cutting angle, even more so than tensile properties.
Impact strength (kJ·m^−2^)	141.1 (S15)	64.2 (S45)	0.545	54.5% of the maximum impact energy absorption is lost. While still significant, this is the lowest anisotropy, suggesting that energy dissipation mechanisms (matrix cracking, fiber pull-out) are less dependent on a single fiber direction and more active across various orientations.

**Table 10:** Predictive equations and goodness of fit for mechanical properties as a function of test orientation angle.

Property	Unit	Expression	R^2^
Tensile strength (TS)	MPa	TS = 106.07 cos^4^(θ) + 156.19 sin^4^(θ) + 22.95 sin^2^(θ)·cos^2^(θ)	0.9605
Tensile modulus (TM)	GPa	TM = 13.38 cos^4^(θ) + 21.01 sin^4^(θ) + 3.70 sin^2^(θ)·cos^2^(θ)	0.9703
Impact strength (IS)	kJ·m^−2^	IS = 137.37 cos^4^(θ) + 92.52 sin^4^(θ) + 119.03 sin^2^(θ)·cos^2^(θ)	0.9712
Flexural strength (FS)	MPa	FS = 111.76 cos^4^(θ) + 65.73 sin^4^(θ) + 439.69 sin^2^(θ)·cos^2^(θ)	0.9459
Flexural modulus (FM)	GPa	FM = 5.59 cos^4^(θ) + 5.29 sin^4^(θ) + 36.21 sin^2^(θ)·cos^2^(θ)	0.9030

## Data Availability

The raw data supporting the conclusions of this article will be made available by the authors on request.
